# The Oral Microbiome Bank of China

**DOI:** 10.1038/s41368-018-0018-x

**Published:** 2018-05-14

**Authors:** Peng Xian, Zhou Xuedong, Xu Xin, Li Yuqing, Li Yan, Li Jiyao, Su Xiaoquan, Huang Shi, Xu Jian, Liao Ga

**Affiliations:** 1 State Key Laboratory of Oral Diseases, National Clinical Research Center for Oral Diseases, West China Hospital of Stomatology, Sichuan University, Chengdu, China; 2 State Key Laboratory of Oral Diseases, National Clinical Research Center for Oral Diseases, Department of Cariology and Endodontics, West China Hospital of Stomatology, Sichuan University, Chengdu, China; 3 Single-Cell Center, CAS Key Laboratory of Biofuels and Shandong Key Laboratory of Energy Genetics, Qingdao Institute of BioEnergy and Bioprocess Technology, Chinese Academy of Sciences, Qingdao, Shandong China

## Abstract

The human microbiome project (HMP) promoted further understanding of human oral microbes. However, research on the human oral microbiota has not made as much progress as research on the gut microbiota. Currently, the causal relationship between the oral microbiota and oral diseases remains unclear, and little is known about the link between the oral microbiota and human systemic diseases. To further understand the contribution of the oral microbiota in oral diseases and systemic diseases, a Human Oral Microbiome Database (HOMD) was established in the US. The HOMD includes 619 taxa in 13 phyla, and most of the microorganisms are from American populations. Due to individual differences in the microbiome, the HOMD does not reflect the Chinese oral microbial status. Herein, we established a new oral microbiome database—the Oral Microbiome Bank of China (OMBC, http://www.sklod.org/ombc). Currently, the OMBC includes information on 289 bacterial strains and 720 clinical samples from the Chinese population, along with lab and clinical information. The OMBC is the first curated description of a Chinese-associated microbiome; it provides tools for use in investigating the role of the oral microbiome in health and diseases, and will give the community abundant data and strain information for future oral microbial studies.

## Microbiology: A census of oral ecosystems

A new resource for consolidating oral microbiome data will help researchers explore the relationship between these commensal communities and the health of their hosts. Numerous studies have highlighted apparent connections between alterations in the microbial communities within the human mouth and medical conditions including diabetes and cancer. A recent article from researchers led by Liao Ga at Sichuan University describes the launch of the Oral Microbiome Bank of China, an effort to study such connections by profiling specimens from individuals from across the country. The database currently houses detailed information on 289 bacterial strains and the samples from which they were obtained. The authors are now looking to analyze these data to gain insights into the structure and function of oral ecosystems, and to further expand this database as a resource for Chinese microbiome research.

## Introduction

With the development of microbiome research in recent years, studies have found that the human microbiome is closely related to systemic diseases.^1–4^ At the end of 2007, the National Institutes of Health (NIH) launched the Human Microbiome Project, which aimed to resolve microorganisms by mapping the genomes of five major parts of the human body (buccal, nasal, vaginal, gut and skin microbiomes).^5,6^ The oral microbial community and oral and systemic health are closely related; they can not only induce dental caries, apical periodontal disease, periodontal disease, pericoronitis, oral mucosal disease and other oral diseases but also many systemic diseases.^7–12^

Based on the clinical features of oral disease diagnosis and treatment, the oral microbiome has more advantages than other parts of the body in terms of convenience, ease of operation and number of patients. Currently, the Human Oral Microbiome Database (HOMD, http://www.homd.org) includes 619 taxa in 13 phyla, 65% of which can be cultured in the laboratory.^13,14^ The human oral cavity is a complex ecological niche, containing both exfoliated surfaces (mucosa) and non-shedding solid surfaces (tooth surfaces), and contains fluid saliva. Therefore, the oral microflora colonised on different surfaces have significant spatial specificity.^15,16^ In the process of individual development and growth, the oral microbiome changes dynamically; as age increases and dentition changes, physiological changes occur in the oral microbiome and the composition of microorganisms in different age groups has large specificity.^2,12,17^ Keijser et al. found that oral microorganisms covered 318 genera of 22 phyla, 5600 and 10,000 of which were colonised in saliva and plaque, respectively.^15,18,19^

As early as 1891, Miller proposed the theory that oral lesion infection may cause a variety of systemic diseases due to oral microorganisms entering other parts of the body through oral infection.^20^ In recent years, with in-depth study of the microbiome, the correlation between the oral microbiome and various systemic diseases has gradually been confirmed, including digestive system diseases, cardiovascular diseases, tumours, premature birth, diabetes and rheumatoid arthritis (RA).^9,21–25^ It has been reported that increased intracellular C-reactive protein levels caused by bacterial infections in the mouth have an important correlation with the development of atherosclerotic vascular disease.^26,27^ C-reactive protein levels in saliva can predict the occurrence of acute myocardial infarction, which will benefit the early diagnosis and control of disease.^28,29^ *Lactobacilli* and *Streptococci*, which are closely related to dental caries, are involved in the pathogenesis of infective endocarditis.^30,31^ Among them, the early colonisation of oral biofilm—*Streptococcus sanguinis* in patients with the highest detection rate of the endocardium—and endocarditis are closely related to the occurrence and development of infective endocarditis.^32,33^ Currently, the pathogenesis occurs with the formation of thrombus-like processes on the surface of normal vascular endothelial cells, promoting bacterial adhesion, which in turn leads to infection.^34,35^ Researchers found that the expression of *Streptococcus gordonii* virulence-associated factor was significantly increased in the process of endocardial infection, which may play a vital role in the endocarditis model.^32,36^ In patients with severe periodontal infection, the oral microbiome interacts with the immune system in a complex manner, producing persistent chronic inflammation, and some oral microbes can also enter the bloodstream.^24,37–39^ A large number of studies have confirmed that diabetes and chronic inflammation in the human body related to periodontitis can lead to systemic inflammatory cytokines, such as cytokines tumor necrosis factor (TNF)-α, interleukin (IL)-1β and IL-6, and can increase the body’s oxidative stress, affecting insulin sensitivity and glucose metabolism.^38,40–43^ Human chronic inflammation caused by periodontal microbial infections is likely to be one of the mechanisms that contributes to the development of diabetes.^15,44–46^ Moreover, a recent study showed that periodontal pathogens involved in the occurrence of RA also exhibit a certain correlation.^47^ *Actinobacillus actinomycetemcomitans* can induce the dysregulation of protein arginine deiminase (PAD) activity in host neutrophils by the secretion of toxin P or toxin A (LtxA), leading to a high degree of citrullination of proteins in neutrophils and the formation of autoantigens.^48^ LtxA also alters neutrophil morphology, mimicking neutrophil extracellular trapping nets and causing neutrophil lysis, eventually releasing massive citrullinated antigens.^43,49^ Unlike *Porphyromonas gingivalis*, which synthesises bacterial PAD, *Actinobacillus actinomycetemcomitans* induces autoantigen formation by inducing endogenous PAD activity, leading to the formation of anti-citrullinated protein antibodies (ACPA) and rheumatoid factor and the development of RA.^30,50^ In addition, the oral microbiome may also serve as a ‘fingerprint’ for the development and treatment of RA.^51^ A recent study found that there is significant ecological imbalance in the oral microbiome of patients with RA, which can be recovered by the treatment of RA. Based on a metagenomic association analysis between the oral and gut microflora, a diagnostic classification model of the human population in the diagnosis of healthy people and RA patients was built with a diagnostic accuracy of nearly 100%, suggesting that the oral microorganism group is linked to the occurrence, development and prognosis of RA.^51^

The human oral microbiome constantly interacts and evolves with human body, and the composition of the human oral microbiome varies greatly in different ethnicities and regions; studying the oral microbiome in the Chinese population is indispensable. Given the importance of the oral microbiome and with the aims to further study the Chinese oral microbiology group and serve oral microbiology researchers in China, the first goal of this study was to develop a provisional taxonomic scheme for unnamed Chinese oral bacterial isolates and phylotypes and provide this information in an online publicly available database, namely, the Chinese Oral Microbiome Bank of China (OMBC) (http://www.sklod.org/ombc). The second goal was to analyse the 16S rRNA gene oral clone sequences to determine the number of clones observed for each Chinese oral taxon and to identify additional taxa that were not included in the initial setup of the OMBC.

## Results

### Overview of the online database

The Chinese Oral Microbiome Database has two main parts currently; one component is bacterial strain information in the Chinese population, which is based on the 16S rRNA gene sequences that were used to define individual Chinese oral taxa and create the taxonomic structures in the database. The other component is clinical sample information, which was obtained through second-generation sequencing and multi-omics analysis to collect biological information on samples for correlation analysis.

**Fig. 1:**
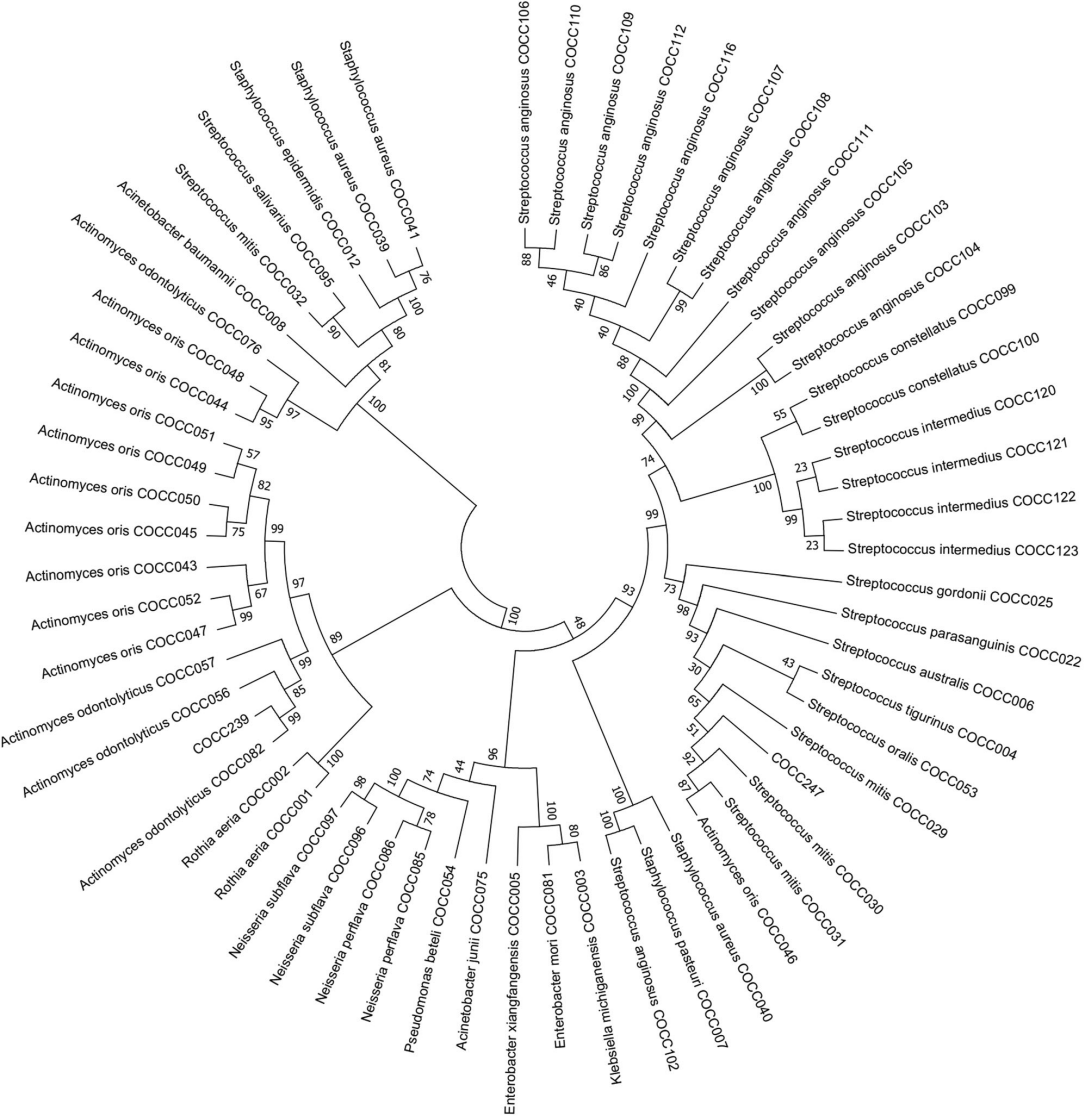
Evolutionary relationships of cultured bacteria. All positions containing gaps and missing data were eliminated. There was a total of 1255 positions in the final data set. Evolutionary analyses were conducted in MEGA7

**Fig. 2:**
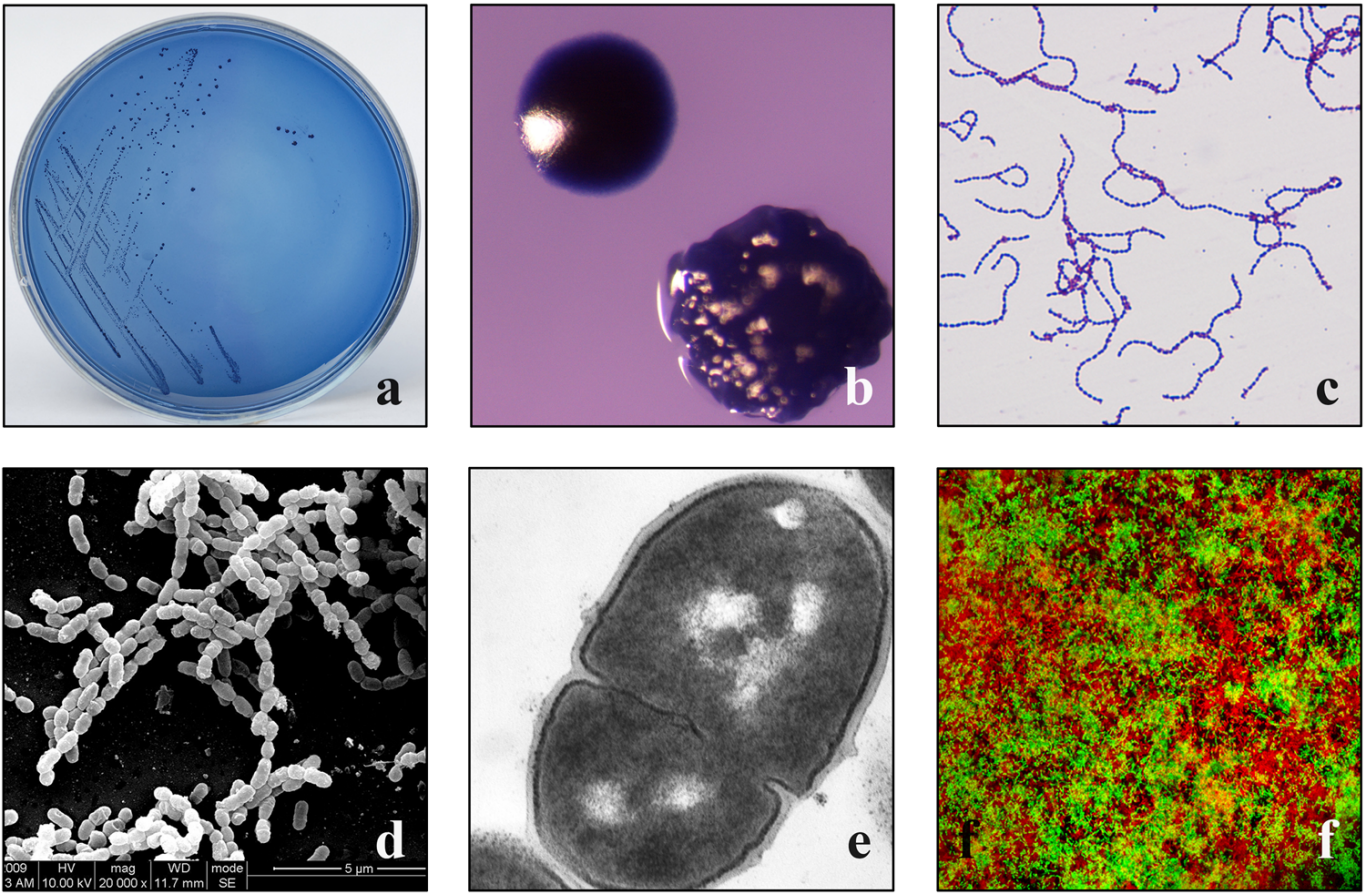
Phenotypic records of a cultured microorganism, a *Streptococcus mutans* strain (COCC139), are shown. **a** Separated microbe on a mitis-salivarius (MS) agar plate. **b** Different colony forms on a blood agar plate. **c** Gram staining. **d** Scanning electron microscopy. **e** Transmission electron microscopy. **f** Observation of extracellular polysaccharides (red) and bacterial cells (green) by confocal laser scanning microscopy

Currently, information on a total of 289 bacterial strains was stored in our online database, and we also collected multi-dimensional characteristics of the bacterial strains by identifying their biochemistry and molecular properties. At the time of writing, the online database contained 60 (20.76%) 16S rRNA-sequenced bacterial strains, 102 (35.29%) of which had biochemical evidence and 117 (40.48%) of which had biochemical descriptions. A detailed exhibition of the phylogenies of the 289 bacteria can be seen in the phylogenetic tree (Fig. 1). The evolutionary tree was inferred using the Neighbour-Joining method.^52^ The bootstrap consensus tree inferred from 1000 replicates was assumed to represent the evolutionary history of the analysed taxa.^53^ Branches corresponding to the partitions reproduced in less than 50% bootstrap replicates are collapsed. The evolutionary distances were computed using the Kimura 2-parameter method^54^ and are in the units of number of base substitutions per site. The name of each bacteria is followed by its designated OMBC accession number. An overview of the phylogenetic distribution of these 289 bacteria is shown in Table 1. Three phyla, Firmicutes, Proteobacteria, Actinobacteria, contain ten families, *Micrococcaceae, Actinomycetaceae*, *Neisseriaceae*, *Enterobacteriaceae, Pseudomonadaceae, Moraxellaceae, Staphylococcaceae*, *Lactobacillaceae, Streptococcaceae* and *Veillonellaceae*. It is remarkable that all the 289 bacteria have been cultured and phenotypically analysed, including their colony colonial morphology, Gram staining, and image recording through a scanning electron microscope, transmission electron microscope and laser confocal microscope (Fig. 2). Furthermore, our database supports views, queries and Basic Local Alignment Search Tool (BLAST), and free downloads of the information on bacterial strains are available. New services, such as a comprehensive analysis system and bacterial strain application system, are under development.

**Table 1:** Phylogenetic distribution of 289 bacteria in the Oral Microbiome Bank of China

Phylum/Number		Class/Number		Order/Number		Family/Number	
NULL	195	NULL	195	NULL	195	NULL	195
*Actinobacteria*	22	*Actinobacteridae*	22	*Actinomycetales*	22	*Micrococcaceae*	3
						*Actinomycetaceae*	19
		*Betaproteobacteria*	5	*Neisseriales*	5	*Neisseriaceae*	5
				*Enterobacteriales*	4	*Enterobacteriaceae*	4
*Proteobacteria*	12	*Gammaproteobacteria*	7	*Pseudomonadales*	3	*Pseudomonadaceae*	1
						*Moraxellaceae*	2
				*Bacillales*	5	*Staphylococcaceae*	5
*Firmicutes*	60	*Bacilli*	57			*Lactobacillaceae*	2
				*Lactobacillales*	52		
						*Streptococcaceae*	50
		*Clostridia*	3	*Clostridiales*	3	*Veillonellaceae*	3

### Web-accessible functions

**Fig. 3:**
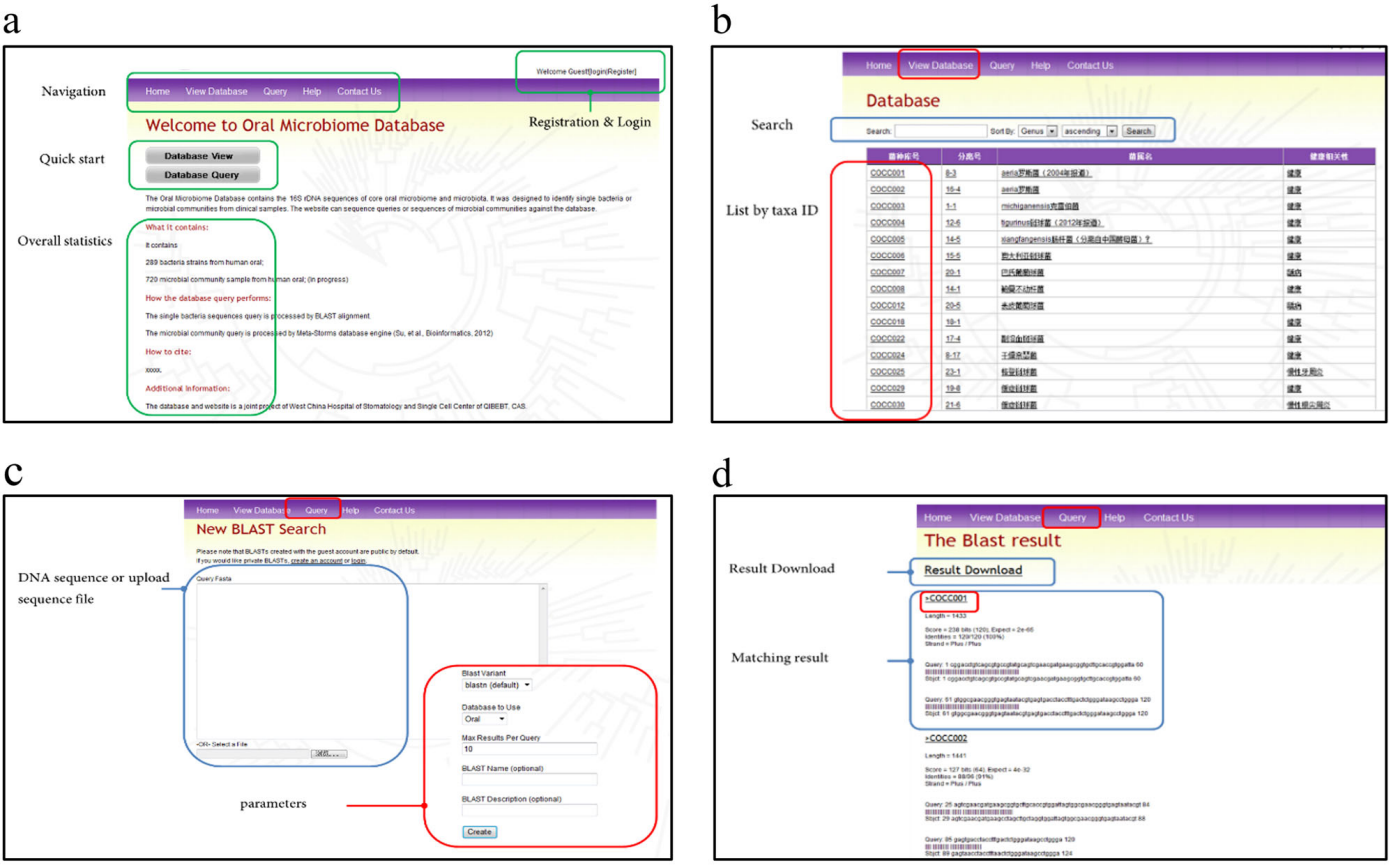
Description of the functions of the database. **a** Layout of the Oral Microbiome Bank of China homepage; **b** Layout of the query and view database page; **c** and **d** BLAST function and result page

The upper navigation menu provides entries for each of the functions of the database. Registration and login functions are located at the upper right. A quick start of the database view and database query are located in the middle of the page, following the overall statistics of the data sets, which is updated real-time (Fig. 3a).

The layout of the view database page contains a search module and list of bacteria by taxa ID. Bacterial information can be searched using a keyword, and the search results can be listed in ascending or descending order by genus, family or order. The results are listed by four main variables, including unique taxa ID, separate no., bacteria name and disease association status. Clicking on the taxa ID or separate ID will lead to the detailed information page of a particular bacterial strain. Clicking each of the keywords (underlined phrase) of the bacterial strain name and health association status column within the result list can trigger a new set of results in the same category with the same keyword. A very convenient function is to determine bacterial strains that have the same characteristics (Fig. 3b). Clicking on the bacteria library ID or separate ID leads to the detailed information page of a particular bacterial strain, and this page displays the most important characteristics of the bacterial strain, which includes the following fields:

Bacteria_Library_Id/Separate_Id: The official library ID and original separate ID of the isolated microorganisms.

Bacteria_Name: The name of the isolated microorganisms in both Latin and Chinese.

Description: Description of the physiological characteristics of the isolated microorganisms.

Kingdom/Phylum/Class/Order/Family/Genus: The official biological classification of the isolated microorganisms.

Separate_Method/Separate_Source: The separate method and source of the isolated microorganisms.

Identify_Method: The method used for the identification of the isolated microorganisms.

Relativity_Healthy: The health status of the individuals from whom the isolated microorganisms were isolated from.

Haemolysis/Gram_Stain/H2O2_Enzyme/Oxidase: The metabolic abilities of the isolated microorganisms.

16S_Sequence/Molecule_Confidence: The sequence of 16S rRNA and the identity of the 16S rRNA sequence.

## Discussion

Currently, aside from traditional microbiome research techniques, many new high-throughput analytical techniques have been developed and adopted in modern microbiome metagenomic studies. We now have a completely new understanding of oral microbes from analysing the characteristics of the oral microbiome samples of twins,^46,55,56^ newborns,^57,58^ infants^59,60^ and children,^61–64^ adolescents,^65,66^ adults,^56,67–69^ and the elderly.^70–72^ The new concept of the ‘oral core microbiome’ suggests that this phenomenon should be personalised in accordance with the ecological characteristics of the oral environment in certain populations at different ages or grades of disease.^15,16,39,73^ By monitoring the dynamic changes in the structure and function of microorganisms, the relationship between oral bacterial flora and these diseases, such as periodontal disease^22,74,75^ and lichen planus,^76,77^ was revealed.

By studying the oral microbiome of patients with systemic diseases, the role of the oral microbiome in the occurrence and development of systemic diseases (leukaemia,^42,78^ head and neck cancer,^79–81^ HIV,^82,83^ diabetes,^42,84^ etc.) was clarified. Chinese researchers also proposed that the microbial indices of caries, the microbial indices of gingivitis (MiG) and the relative microbial recovery indices were used to evaluate gingival health care programmes based on the distribution pattern of bacteria in different sites within the oral cavity. The potential role of oral microbes in the diagnosis and prognosis of systemic diseases was demonstrated through applications in studies on RA.^50,85^

The oral cavity is an interactive environment. Oral diseases, such as oral lichen planus and oral cavity cancer, are closely related to oral microorganisms. Therefore, it is of great significance for multi-factor studies on oral diseases to construct a comprehensive oral microbial database. By combining the clinical data of oral microbial communities and the microbiological metagenomics data of healthy and Chinese patients from multiple regions, ethnicities and ages, the OMBC was established, and clinical samples were collected, isolated and identified. The OMBC is the first publicly available HOMD for the Chinese population. Moreover, the OMBC has several key features. First, this well-organised database was built according to industrial standards for maximum expansion and migration capacity, and regulations regarding microbial data management were established. Second, the OMBC contains all kinds of metadata, such as 16S rRNA sequencing data and key property information on the microbiome, and can compare bacterial 16S rRNA sequences and predict oral microbial classifications and related clinical information based on clinical sample gene sequences. Third, the taxa ID that we created can be used as a unique identifier of the Chinese oral microbiome to facilitate connections and communication among different studies. Fourth, all data can be filtered and sorted in many precise methods to maximise query efficiency. Lastly, all of the data can be downloaded freely via our website.

However, there are still many limitations of our database. The current quantity of the microbiome is to be improved as more samples and data are collected continuously. Additional registries will make this database more useful for future multi-centre studies and will more accurately reflect the overall distribution and evolution trends of the microbiome of the Chinese population. In addition, we also plan to expand our data dimension by adding more detailed data and external data in addition to the current data sets. In the meantime, we will improve data quality.

To improve the visibility and usability of the OMBC, we are working to carry out extensive big data studies with the database to obtain more profound insights into the oral microbiome, such as interaction networks among bacteria. It is also our mission and responsibility to build this database into a national platform as an important component of the world microbiome field and to benefit global microbiome investigators. The characteristics and commonality of microbiome-related phenomena are receiving more and more attention. Understanding microbiomes closely related to humans helps us better understand human beings, and comprehensive studies on the microbiome help with the prevention, diagnosis and treatment of many major human diseases in modern precision medicine.

## Materials and Methods

### Collection and transport of samples

#### Collection of saliva samples

Unstimulated salivary samples were collected as described previously, which was followed by a gentle rinse with warm water to remove the food residue. In addition, 0.5–1.0 mL of naturally secreted saliva was collected, or saliva samples in the oral cavity were absorbed directly by the sterile pipette tips of a micro-concentrator.

#### Collection of plaque samples

Plaque samples were collected in different ways depending on different clinical requirements and purposes. Plaque indicators were used to display plaque for some collections. Before sample collection, subjects gargled with warm water to remove food residue in their mouths. Then, sterile gauze or a yarn ball was used to isolate saliva and collect plaque samples or decayed materials. A sterile probe was used in the collection of plaque in the occlusal surface fissure. Adjacent surface plaque samples were collected with both a sterile probe and dental floss or with a fine wire used in orthodontics. A sterile curette was used in the collection of root surface plaque samples. Plaque samples on the gum or gingival margin were collected by a spoon scaler.

#### Collection of other infection samples

Infection samples were generally sampled with sterile cotton swabs. A purulent fluid sample of the periodontal abscess was collected with sterile syringes. The tissue pieces in the alveolar socket were generally collected as samples of dry sockets developed after tooth extraction.

#### Collection of oral mucosal diseases samples

White membrane materials were collected with curettes or cotton swabs. To collect quantitative samples, filter papers with a particular area are used.

#### Sample delivery

Among oral clinical specimens, the detection of specimens of fungus, aerobic bacteria and general facultative anaerobic bacteria were cotton swab-sampled and sent in sterile tubes directly. For the detection of most anaerobic bacteria or microaerophilic bacteria, samples were sent to the laboratory as soon as possible in an anaerobic way. In addition, a pus or saliva sample was inserted directly into the needlepoint sterile rubber stopper of a syringe needle tube for transport; most of the samples were put in the prereduction of anaerobic culture media and transported to the lab after immediately acquisition in order to reduce the death of bacteria that were sensitive to oxygen in the carrying process. Vaccination beside the chair and anaerobic delivery were used to improve the detection rate of obligate anaerobic bacteria. For clinical specimens that could not be inspected in a timely manner or delivered over a long distance, anaerobic bags (commercially available) or prereductions of liquid spiral tubules stamped with liquid paraffin were used for delivery.

### Dispersion and dilution of samples

Oral clinical infection is generally a mixed infection of many bacteria, with mixed species and varying quantities of bacteria in a concentrated plaque mass. Thus, an oral clinical specimen is usually required for inoculation after decentralised processing and dilution to achieve a single colony of pure culture.

#### Sample dispersion

Generally, two methods, named spiral vortex oscillation and ultrasonic dispersing, are adopted.

#### Sample dilution

Oral clinical samples are mixed bacterial infection samples, with mixes in the number and variety of bacteria. Therefore, proper concentrations of diluent dilution are required before vaccination to obtain a single colony after the sample dispersion process. The transporting fluid can be used as a diluent, and a phosphate buffer with a pH value of 7.2 is also available. Ten-times dilution series are usually used. Under an aseptic operating status, a specimen liquid of 0.1 mL is added to 0.9 mL of diluent, and 0.1 mL of mixture after the diluent is thoroughly incorporated (10^−1^) is added to a tube containing 0.9 mL of diluent for blending. According to the above method, 10-times dilution series are achieved. Due to differences in sample bacteria, different samples have different diluted concentrations, such as a saliva sample dilution degree of 10^−4^~10^−6^, a gingiva groove plaque dilution of 10^−1^~10^−2^ and a plaque collection dilution of 10^−3^~10^−5^.

### Inoculation and incubation of samples

In addition, to select the appropriate medium before inoculation, vaccination dilution degrees, inoculation method and incubation environment, and times must be established according to the specimen type, purpose and microbial species.

#### Selection of the medium

The basic media commonly used for oral bacteria include cardio-cerebral immersion medium, pancreatic enzyme-hydrolysed soy agar (Trypticase soy agar) and Tryptone yeast extract agar medium. These media can be used to cultivate most bacteria in oral samples. Approximately 5% of fibre blood serum (or 5% serum) and chlorinated haemoglobin and vitamin K1 were supplied to the culture medium for some obligate anaerobic gram-negative bacterium. To train general aerobic and facultative anaerobic bacteria, ordinary medium containing blood agar was used.

#### Inoculation of samples

The spread method, drop method and spiral vaccination method were adopted for oral clinical bacteriology samples, which makes the appropriate dilution degrees of the specimen solution and quantitative inoculation on the agar plate.

#### Incubation of samples

For a medium that has received clinical samples, its incubation conditions are determined according to the requirements of cultivation, including atmospheric conditions, temperature and time. Oral clinical specimens, such as an infected root canal, pericoronitis infection after tooth extraction and samples under the gums of periodontitis plaque, whose main characteristics are mixed bacterial infection, are generally involved different atmospheric conditions with a variety of microorganisms with their respective characteristics. An anaerobic culture containing 80 N_2_, 10 CO_2_ and 10% H_2_ at atmospheric conditions with a temperature of 37 °C and a time of 48–72 h was used to grow the bacteria. Some bacteria in the mouth, such as *Treponema*, were trained in the anaerobic environment for approximately 1 week. Common anaerobic incubation devices include an anaerobic glove box, anaerobic incubation and anaerobic bag.

### Smear and stain

Smear test and slide stain are basic techniques for the identification of microbes, and they are primarily used for morphological observation. A combination of smear and stain tests is widely used in oral microbial research for differentiating spirochaete, bacteria, fungi and protozoa and to identify specific cellular structures, including spores, capsules and flagella. Therefore, we used a direct smear test and stained smear test under a microscope for cellular morphological examination, including gram staining and Congo red staining.

### Growth characteristics and identification

Phenotypic organism identification is the most basic and important part of microbiology. The classical method for bacterial identification is to observe the phenotypic characteristics on a foundation of pure bacterial culture, including colony characteristics (size, colour, shape, etc.), cell characteristics (size, shape, arrangement and dying), special structure (with or without spores, capsule and flagellum), culture characteristics (sensitivity to oxygen, optimum growth temperature and pH, requirements for nutrients and growth factors, etc.) and metabolites. Berkey’s Manual of Systemic Bacteriology, which is an authoritative reference book for bacterial isolation, is the reference for the growth characteristics of bacteria. Bacterial colony morphology includes size, colour, shape, growth patterns and characteristics. Haemolytic reaction is one of the basic characteristics of bacterial identification. Some bacteria can produce a haemolytic reaction, whereas some produce pigmentation; this test has been used for differentiation; some bacteria produce gas and some bacteria exhibit their own growth patterns, such as the migration of Proteus growth.

### Biochemical tests

Biochemical tests are an important microbial identification method. Routinely, biochemical tests include the carbohydrate fermentation test, methyl red test, citric acid utilisation test and hydrogen sulphide production test. The micro-biochemical test reaction plate we used included 30 biochemical matrixes and relevant biochemical test indicators, phosphate-buffered saline, a bacterial turbidity standard tube and eight identification series and was used in a VITEK-2 COMPACT.

### Molecular method

The 16S rRNA gene was used as the standard for the classification and identification of microbes because it is present in most microbes and shows proper changes. Type strains of 16S rRNA gene sequences for most bacteria and archaea are available in public databases (GenBank).

### DNA extraction and sequencing

Total DNA was extracted from collected samples from each respective host. The barcoded 16S rRNA amplicons^86^ (V1-V3 hypervariable region) of all samples were sequenced on using a Roche 454 FLX Titanium. Pyrosequencing data were analysed using scripts from MOTHUR,^87^ QIIME,^88^ and custom R scripts. All raw sequences were deposited at the OMBC.^89^

### 16S rRNA alignment

All bacterial 16S rRNA gene sequences that we believe represent oral taxa and named human oral species in GenBank were entered. Evolutionary analyses were conducted by exporting aligned sequences from our database in MEGA7.^90^ Phylogenetic trees were made using the Neighbour-Joining method.^52^ Bootstrapping was performed using 1000 replicates.^53^

### Database and web design

**Fig. 4:**
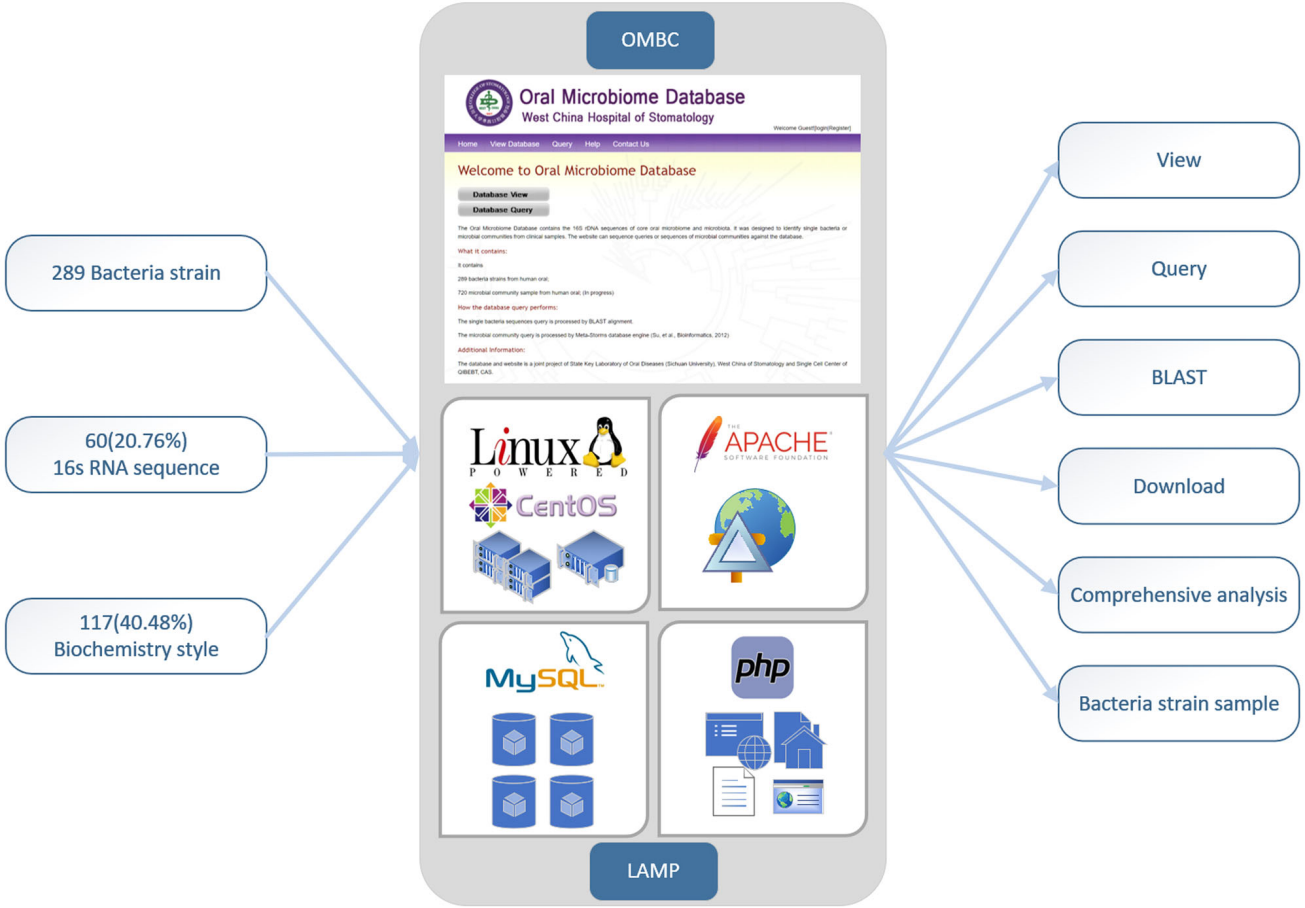
Description of the essential elements and main methodology used in the establishment of the OMBC. The backbone of the database is the LAMP model

The backbone of our platform is an industrial standard LAMP system (Fig. 4). Linux (CentOS) provides maximum stability and a multithread computation environment as the operating system; Apache provides the most important and fundamental function as the web service; MySQL works as the relational database, and PHP is adopted for dynamic web page rendering. PHP is also used to code the common gateway interface to the relational database. Our database can be accessed via the URL: http://www.sklod.org/ombc

### Curation

The project investigators P.X., Z.X., X.X., L.Y., L.Y., L.J., S.X., H.S., X.J. and L.G. carried out the curation of the database. These investigators reviewed each item on the taxon description page.

### Service and function

The database and query and statistical functions deployed were designed and developed according to the suggestions of more than 20 professionals in microbiome research with more than 10 years of expertise in this field. The key features of our database include the following. (1) The database has been designed with a user friendly interface. Users can start using the core functions immediately without professional training. (2) The back-office management system provides add, delete and modify record functions for administrators. We plan to make the system a public platform that enables users to upload their own bacterial strain information in the near future. (3) Query provides statistical functions that can efficiently analyse the trends and distributions of each variable of the data set as a whole. (4) Export and backup functions plus a complete restoration mechanism ensure data security and integrity.
